# Effect of selective gastric residual monitoring on enteral intake in preterm infants

**DOI:** 10.1186/s13052-022-01208-7

**Published:** 2022-02-17

**Authors:** Serena Elia, Martina Ciarcià, Francesca Miselli, Giovanna Bertini, Carlo Dani

**Affiliations:** 1 Division of Neonatology, Careggi University Hospital of Florence, Florence, Italy; 2 Department of Neurosciences Psychology Drug Research and Child Health, Careggi University Hospital of Florence, Florence, Italy

**Keywords:** Gastric residual, Feeding intolerance, Necrotizing enterocolitis, Preterm infant

## Abstract

**Objective:**

Prefeed gastric residuals (GRs) monitoring has been correlated with an increased time to reach full feeds and longer parenteral nutrition without beneficial effect on necrotizing enterocolitis (NEC) occurrence. We aimed to assess effects of a new local protocol to provide for the selective evaluation of GRs excluding their routine monitoring.

**Methods:**

We carried out a retrospective study based on a “before and after” design in a cohort of infants born at 23^+0^–31^+6^ weeks of gestation. The primary outcome was the age at full enteral feeding (150 mL/kg/d). Secondary outcomes included age at regaining of birth weight, and evaluation of Z-scores of weight, length, and head circumference at discharge.

**Results:**

We studied 49 infants in the selective GR group and 59 in the routine GR group. Age at full (150 mL/kg) enteral feeding (17.8 ± 10.1 vs. 22.9 ± 10.5 days, *P* = 0.017) and regaining of birth weight (11.1 ± 3.0 vs. 12.5 ± 3.5 days, *P* = 0.039) were lower while the Z-scores of weight at discharge (-1.10 ± 0.83 vs. -1.60 ± 1.45, *P* = 0.040) were higher in infants in the selective GR group in comparison with infants in the routine GR group.

**Conclusions:**

Selective monitoring of GRs decreased age at full enteral feeding and at regaining of birth weight and induced better Z-scores of weight at discharge in comparison with routine GR monitoring in a cohort of extremely preterm infants without increasing the incidence of NEC. Omitting prefeed GRs monitoring in clinical practice seems reasonable.

**Supplementary Information:**

The online version contains supplementary material available at 10.1186/s13052-022-01208-7.

## Background

Gastric residuals (GRs) evaluation consists in measuring the volume of milk along with gastrointestinal secretions remaining in the stomach after a certain time interval [1]. It is common to find GRs in preterm infants due to the immaturity of the gastrointestinal tract in the terms of decreased length, immature motility patterns, and inadequate digestive and absorptive capacity [2, 3]. It has been postulated that the negative pressure created by repeated aspiration of gastric contents might damage the fragile gastric mucosa, and discarding the gastric contents results in a loss of gastric enzymes and gastric acid in preterm infants. However, the use of GRs as a marker of feeding intolerance [4] is widespread in neonatal intensive care units (NICUs) and has contributed to making a diagnosis of feeding intolerance very frequent. It has been estimated that 16 to 29% of premature infants admitted to NICUs develop a feeding intolerance [5, 6] whose subsequent management depends on nurses’ experience, clinicians’ individual preference, and NICU protocols. On the other hand, feeding intolerance has been associated with an increased risk of developing necrotizing enterocolitis (NEC) [7, 8] and, therefore, an increased volume of altered GRs (bilious- or blood-stained) has often, in turn, been considered a risk factor for the development of NEC. Thus, the importance and diffusion of GRs (and feeding intolerance) monitoring depends on its possible usefulness in the prevention or early diagnosis of NEC, which remains the most frequent neonatal gastrointestinal complication in preterm infants [9, 10] increasing their mortality ^8^ and risk of neurodevelopment delay [11]. Nevertheless, the role of prefeed GRs monitoring to detect feeding intolerance (and a potential risk of NEC) is controversial and has been correlated to an overestimation of feeding intolerance increasing the number of feed interruptions and the time taken to reach full feeds without beneficial effect on the occurrence of NEC [1, 12, 13]. These results are relevant because achievement of full enteral feeding allows better preterm infant nutrition and growth, prompt discontinuation of parenteral nutrition and reduction of catheter-related bloodstream infections [1, 14]. Based on these considerations, we decided to implement a new local protocol in our NICU that provides for selective rather than routine evaluation of GRs with the assumption that this might allow earlier full enteral feeding to be achieved and improvement of patients’ growth. Thus, the aim of this study was to evaluate the effects of the new strategy on these outcomes in comparison to the previous approach.

## Methods

### Patient population

This center-based study was carried out at the NICU of Careggi University Hospital in Florence, Italy. Infants were included in the study if they were born at 23^+0^–31^+6^ weeks of gestation. Exclusion criteria were major congenital malformations, chromosomal disorders, inherited metabolic diseases, and death before reaching full enteral feeding (150 mL/kg).

### Study design

The new local protocol for GRs monitoring was introduced in October 2020 and routine evaluations of GRs before every feeding were discontinued. Patients undergoing selective GRs monitoring were prospectively studied from December 2020 to June 2021, while patients in the routine GRs monitoring group were retrospectively studied from December 2019 to June 2020.

Evaluation of GRs in the selective group was performed in case of established (positive blood culture) or suspected sepsis (pathological values of C-reactive protein (CRP) and consistent clinical signs and symptoms), NEC at stage 1 or higher [15], established or suspected gastrointestinal occlusion, ascites, and established feeding intolerance. The latter was determined by abdomen pathological physical examination, regurgitations/vomits, GRs with volume ≥ 100% of previous feed and/or altered (i.e.: hematic, fecaloid), mucous or bloody stools, and onset of apnea/bradycardia [16]. The severity of these conditions was graded as minor and major criteria of feeding intolerance and were used for deciding the interruption of enteral feeding: the occurrence of one to two minor criteria suggested no change in the volume of feed, while the occurrence of three minor criteria or one major criterion suggested discontinuation of enteral feeding [16]. (Table S1, supplemental materials) Evaluation of GRs in the routine group was performed before every feeding and the decisions to increase, reduce, or halt feeding were made based on the same criteria ^17^.

All infants followed the same enteral nutrition protocol: trophic feeding was initiated within 24 h after birth and continued at 20–40 mL/kg/d as tolerated for up to five days. Subsequently, the amount was increased by 20 ml/kg per day if enteral nutrition was tolerated. Enteral nutrition goals were 150 mL/kg/d and 120 kcal/kg/d. All patients received their mother’s or donor’s human milk administered through an oro-gastric tube as bolus or continuous milk feeding. Human milk was enriched with a fortifier (Prenidina FM85®, Nestlè, La Tour-de-Peilz, France; 1 g/25 mL of milk) when enteral feeding of 100 ml/kg/d was reached. Preterm formula was administered when human milk was not available. Parenteral nutrition was continued until feeds of 100 mL/kg/d were reached.

### Study outcomes

The primary outcome of this study was age at full enteral feeding (150 mL/kg/d). Prespecified secondary outcomes included age at full oral (breast and bottle) enteral feeding and at birth weight recovery, duration of parenteral nutrition, stay in NICU and in hospital, and evaluation of Z-scores of weight, length, and head circumference at discharge.

### Data collection

Clinical and demographic data were collected by reviewing patients’ electronic medical records. The following data were recorded for each studied infant: gestational age, birth weight, birth weight < 10^th^ percentile, antenatal steroids, mode of delivery, need and duration of non-invasive and invasive ventilation, surfactant and postnatal steroid treatment, occurrence of patent ductus arteriosus (PDA) requiring treatment, bronchopulmonary dysplasia (BPD), necrotizing enterocolitis (NEC), sepsis, intraventricular hemorrhage (IVH), retinopathy of prematurity (ROP), mortality, duration of stay in NICU and in hospital, and occurrence of breastmilk (exclusive or mixed) feeding at discharge. BPD was defined as oxygen requirement at 36 weeks of post-menstrual age. PDA was diagnosed by echocardiography; NEC was defined as Bell’s stage ≥ 2 [15] sepsis was defined as positive blood culture. IVH was classified according to the Papile classification scheme [17]; ROP was graded according to the international classification of retinopathy of prematurity [18]. The examined maternal variables included clinical chorioamnionitis, maternal diabetes, hypertensive disorders of pregnancy, and prolonged premature rupture of membranes (pPROM) > 18 h. Z-scores of weight, length, and head circumference at discharge were calculated using Anthro version 3.2.2 (World Health Organization, Geneva, Switzerland) [19]. Growth Z-scores are normed for age and sex to provide a more meaningful description of postnatal growth than raw values.

### Statistical analysis

Clinical characteristics of infants in the selective and routine GRs monitoring groups were reported as mean and standard deviation, rate and percentage, or median and range. Statistical analysis was performed using the Student “t” test for parametric continuous variables, the two sample Wilcoxon rank-sum test for non-parametric continuous variables, and the Fisher test for categorical variables. A *p* < 0.05 was considered statistically significant.

We planned to perform logistic regression analyses to assess the possible correlation between the primary outcome and variables that at univariate analysis were different between the groups (*p* < 0.100) excluding those which were found to be collinear by calculating variance inflation factors (VIF). However, there were not significant differences between the no residual and residual groups and logistic regression analysis was not required. Post-hoc analysis demonstrated that our study had 73% power to detect as statistically significant the observed differences of age at full enteral feeding between the groups.

## Results

A total of 118 infants were enrolled in the study, 49 in the selective GR group and 59 in the routine GR group. Clinical characteristics, including prematurity complications and particularly the occurrence of NEC (6 vs. 2%; *P* = 0.320), were similar between the groups (Tables 1 and 2).

**Table 1:** Demographic and clinical characteristics of infants in the selective and routine gastric residual groups. Mean (± *SD*), median (range), or rate (%)

	Selective Gastric Residual Group (*n* = 49)	Routine Gastric Residual Group (*n* = 59)	*P*
Gestational age (wks)	27.8 ± 2.2	28.1 ± 2.5	0.537
Birth weight (g) < 10° percentile	1084 ± 347 5 (11)	1100 ± 427 9 (15)	0.843 0.572
Antenatal steroids	44 (94)	50 (85)	0.219
Vaginal delivery	22 (47)	35 (59)	0.241
Apgar Score at 5 min	8 (3–9)	8 (6–9)	1.000
Noninvasive ventilation	42 (89) 21.6 ± 18.5	50 (85) 22.0 ± 18.1	0.572 0.911
Mechanical ventilation Duration (d)	13 (28) 18.2 ± 22.1	19 (32) 17.1 ± 19.9	0.674 0.788
Surfactant	23 (49)	36 (61)	0.242
Postnatal steroids	9 (19)	15 (25)	0.491
Patent ductus arteriosus	22 (47)	31 (53)	0.696
Bronchopulmonary dysplasia	18 (38)	27 (51)	0.446
Necrotizing enterocolitis	3 (6)	1 (2)	0.320
Sepsis	9 (19)	12 (20)	1.000
Intraventricular haemorrhage	6 (13)	14 (24)	0.212
Retinopathy of prematurity	1 (2)	5 (8)	0.224
Death	3 (6)	5 (8)	0.491
Pregnancy complications			
Chorioamnionitis	3 (6)	11 (19)	0.085
Gestational diabetes	8 (17)	6 (10)	0.392
Hypertensive disorders of pregnancy	7 (15)	4 (7)	0.210
pPROM > 18 h	11 (23)	20 (34)	0.286

pPROM: prolonged premature rupture of membranes

**Table 2:** Weight, length, and head circumference at birth and discharge and type of feeding at discharge in infants in the selective and routine gastric residual groups. Mean (± *SD*)

	Selective Gastric Residual Group	Routine Gastric Residual Group	*P*
Birth weight (g) Z-score	1084 ± 347 0.25 ± 0.89	1100 ± 427 -0.07 ± 1.05	0.843 0.164
Weight at discharge (g) Z-score	2422 ± 609 -1.10 ± 0.83	2536 ± 611 -1.60 ± 1.45	0.362 0.040
Birth length (cm) Z-score	37.3 ± 2.8 -0.47 ± 1.87	36.4 ± 4.3 -0.66 ± 2.39	0.913 0,715
Length at discharge (cm) Z-score	44.2 ± 3.5 -1.60 ± 1.45	44.9 ± 4.6 -2.12 ± 1.28	0.409 0.138
Birth head circumference (cm) Z-score	26.6 ± 2.3 0.01 ± 1.00	25.9 ± 2.9 -0.61 ± 2.13	0.197 0.144
Head circumference at discharge (cm) Z-score	31.5 ± 2.5 -1.27 ± 0.53	32.6 ± 3.0 -1.14 ± 2.32	0.056 0.796
Breastmilk at discharge: Exclusive Mixed None	15 (33) 10 (22) 21 (46)	17 (31) 17 (31) 20 (37)	1.000 0.367 0.543

Age at full (150 mL/kg) enteral feeding (17.8 ± 10.1 vs. 22.9 ± 10.5 days, *P* = 0.017), full oral enteral feeding (56.2 ± 6.1 vs. 58.3 ± 4.0 days, *P* = 0.045), and birth weight recovery (11.1 ± 3.0 vs. 12.5 ± 3.5 days, *P* = 0.039) was lower while Z-scores of weight at discharge (-1.10 ± 0.83 vs. -1.60 ± 1.45, *P* = 0.040) were higher in infants in the selective GR group in comparison to infants in the routine GR group (Tables 2 and 3). We observed a trend toward shorter duration of parenteral nutrition, stay in NICU and in hospital in the no routine residual group in comparison with controls, but differences did not reach statistical significance (Table 2). Exclusive and mixed breastmilk feeding at discharge had similar occurrence in the groups (Table 2).

**Table 3:** Primary and secondary outcomes in infants in the selective and routine gastric residual groups. Mean (± *SD*)

	Selective Gastric Residual Group (*n* = 46)	Routine Gastric Residual Group (*n* = 54)	P
Age at full (150 ml/kg/die) enteral feeding (d)	17.8 ± 10.1	22.9 ± 10.5	0.017
Age at full oral enteral feeding* (d)	56.2 ± 6.1	58.3 ± 4.0	0.045
Age at birth weight recovery (d)	11.1 ± 3.0	12.5 ± 3.5	0.039
Duration of parenteral Nutrition (d)	18.1 ± 15.4	23.0 ± 14.3	0.108
Duration of NICU stay (d)	35 ± 21	40 ± 31	0.365
Duration of hospital stay duration (d)	65 ± 27	70 ± 42	0.497

NICU: neonatal intensive care unit, *Breast and bottle feeding

## Discussion

In this study we evaluated the effects of implementing in our NICU a new protocol for the monitoring of GRs through a selective monitoring approach rather than routine monitoring. We found that the new procedure allowed achievement of full enteral feeding and full oral (breast and bottle) enteral feeding sooner. These results are in agreement with previous studies. Abiramalatha et al. meta-analysed two randomised controlled studies which enrolled 141 very low birth weight infants and found that routine monitoring of GRs increases the time needed to establish full enteral feeds, to regain birth weight, and the s duration of parenteral nutrition [1]. Kumar et al. meta-analysed six studies which enrolled 451 preterm infants and found that avoidance of routine prefeed evaluation of GRs was associated with earlier achievement of full enteral feeding, shorter duration of hospitalization, and lower incidence of late-onset sepsis [20]. These results are explained by the inappropriate discontinuation of enteral feeding with subsequent delays in advancement of enteral nutrition associated with routine prefeed assessment of GRs. In fact, it is well known that GR evaluation is not an accurate marker of feeding intolerance due to a lack of standardization regarding evaluation of gastric tube placement, also in relation to patient position [20], the most appropriate timing of assessment, the volume and/or color that should be considered abnormal and, moreover, the use of GRs which could be returned or discarded [21].

Our study confirms that avoiding routine monitoring of GRs is not associated with an increase of incidence of NEC. A similar result was reported by several studies [12, 13, 21, 22] and meta-analyses [1, 23] and, more recently, by Purohit et al. who found a lack of association between GRs and the risk of developing a NEC ≥ stage II [24]. These findings are consistent with the study by Mihatsch et al. who concluded that assessment of GRs (volume and color) is not only not predictive of feeding intolerance, but also not predictive of NEC [25]. Other studies [26, 27] have reported, in contrast, an association between increased GRs and NEC, but they were retrospective and had many confounding factors which limit their value. Thus, GRs alone are an unreliable marker of NEC, while in association with other suspicious clinical signs [16] they can contribute to a rational management of enteral nutrition and to the planning of any diagnostic tests.

We found that the time to regain birth weight and Z-scores of weight at discharge were significantly better in infants in the no routine residual group than in the control group, while Z-scores of length and head circumference were similar. These findings are in agreement with previous studies [1, 12, 13] and confirm the positive effect on nutrition and growth of a selective GRs evaluation policy which accelerates the time needed to reach full enteral feeding and improves the nutritional intake of these infants.

The main limitation of this study is that it was a retrospective single center study based on a “before and after” design which cannot rule out the possibility of unmeasured confounding factors contributing to the results. However, during the study periods clinical practice at the center did not change and our findings were substantially in agreement with previous studies. These considerations, together with the similar demographic and clinical characteristics of the study groups, support the reliability of our results. Another limitation regards the size of our population which did not allow us to find statistical significance in the shortening of parental nutrition, stay in NICU and in hospital duration that we observed as a trend in the selective GR monitoring group in comparison with the routine GR monitoring group. Furthermore, although we have not observed significant changes in the onset of NEC, our study does not have sufficient power to definitively exclude the risk of its increase. However, despite these limitations, we believe our study adds useful contemporary data about implementing a new protocol of monitoring in term of its effects and safety in a NICU.

## Conclusions

In conclusion, we found that the selective monitoring of GRs in a cohort of extremely preterm infants was associated with a decrease of age at full enteral feeding and at birth weight recovery and with better Z-scores of weight at discharge in comparison with routine GR monitoring. The incidence of NEC did not increase although this finding, while in agreement with previous studies, should be confirmed in a larger population. The assessment of GRs is time consuming and devoid of beneficial effects. Thus, its current widespread application seems no longer justified according to literature evidence, while it seems reasonable to omit prefeed GRs monitoring in clinical practice.

## Supplementary Information

**Additional file 1.**

## Data Availability

The datasets used and/or analysed during the current study are available from the corresponding author on reasonable request.

## Abbreviations

BiPAP: Bi-level NCPAP
BPD: Bronchopulmonary dysplasia
GRs: Gastric residuals
HFNC: High-flow nasal cannulae
IVH: Intraventricular hemorrhage
NEC: Necrotizing enterocolitis
NICUs: Neonatal intensive care units
Pprom: Prolonged premature rupture of membranes
ROP: Retinopathy of prematurity
VIF: Variance inflation factors
