# Supplementary material for: Effect of selective gastric residual monitoring on enteral intake in preterm infants
Source: Ital J Pediatr. 2022 Feb 17;48:30. doi: 10.1186/s13052-022-01208-7 (PMC8851703; doi:10.1186/s13052-022-01208-7)
Supplement: Supplementary file 1 — Additional file 1. [file 13052_2022_1208_MOESM1_ESM.docx]

**Table S1.** Minor and major criteria for feeding intolerance diagnosis and their interpretation. Modified from Cresi et al. ^17^.

|  | **Minor criteria** | **Major criteria** |
| --- | --- | --- |
| **Physical examination** | Abdominal distension.  Visible bowel ansa.  Abdominal distension responsive to gastric suction/rectal stimulation. | Dyschromic abdominal wall.  Abdominal distension not responsive to gastric  detension/rectal stimulation.  Painful abdomen. |
| **Regurgitations/vomits** | ≤ 2 episodes between 2 feeds or in the previous 3 h (if not fed). | Episodes between 2 feeds or in the previous 3 h (if not fed).  Bilious vomiting/hematemesis |
| **Gastric residual volumes and color** | <100% of previous feed (bilious or with hematic fragments). | Hematic/fecaloidal.  ≥100% of previous feed. |
| **Alvus** | Mucous stools. | Hematic stools. |
| **Cardiorespiratory (CR) events** | ≥3 CR events/h *. | ≥1 extreme CR event **. |
| **Interpretation** | | |
| 0–1 minor criterion:  Continue enteral feeding with increments | | |
| 2 minor criteria:  Stop increasing feeds, reassess prior to the next to the next feed, and evaluate GRs if not done before.  If 2 minor criteria in at least 2 consecutive evaluations, consider reducing volume of feed. | | |
| 1 major criterion or 3 minor criteria:  Interrupt enteral feeding and re-assess prior to the next feed. | | |

*CR events were defined as episodes of apnea lasting more than 20s or more than 5s if followed by desaturation or bradycardia, episodes of desaturation with blood oxygen saturation <80%, and episodes of bradycardia with heart rate <80 beats/min.

**Extreme CR events were defined as CR events requiring resuscitation.
